# Nitric Oxide: A Regulator of Cellular Function in Health and Disease

**DOI:** 10.1155/2016/9782346

**Published:** 2015-12-21

**Authors:** Luis Sobrevia, Lezanne Ooi, Scott Ryan, Joern R. Steinert

**Affiliations:** ^1^Cellular and Molecular Physiology Laboratory (CMPL), Division of Obstetrics and Gynaecology, School of Medicine, Faculty of Medicine, Pontificia Universidad Católica de Chile, 8330024 Santiago, Chile; ^2^Department of Physiology, Faculty of Pharmacy, Universidad de Sevilla, 41012 Seville, Spain; ^3^University of Queensland Centre for Clinical Research (UQCCR), Faculty of Medicine and Biomedical Sciences, University of Queensland, Herston, QLD 4029, Australia; ^4^School of Biological Sciences, Illawarra Health and Medical Research Institute, University of Wollongong, Northfields Avenue, Wollongong, NSW 2522, Australia; ^5^Department of Molecular and Cellular Biology, University of Guelph, 50 Stone Road E., Guelph, ON, Canada N1G 2W1; ^6^MRC Toxicology Unit, Lancaster Road, Leicester LE1 9HN, UK

Nitric oxide (NO) is a gaseous messenger molecule synthesized from L-arginine and molecular oxygen by three different NO synthases, that is, neuronal (nNOS), endothelial (eNOS), and inducible (iNOS) form [1]. Since its discovery in the early 1980s by the three Nobel Laureates Furchgott, Ignarro & Murad [2], NO has been widely recognised as an important signalling molecule in many physiological processes. The initial identification of NO as an endothelium-derived relaxing factor (EDRF) [3] generated great interest in its function in vascular biology. Over the following years, however, the focus on NO research rapidly expanded from the vascular system to its role in immunity and inflammation, the nervous system, pregnancy, aging, and cell death.

Many studies suggest that excessive or abnormal production of NO plays a crucial role in neuronal cell death associated with neurodegenerative disorders such as Alzheimer's and Parkinson's diseases, as well as various conditions of vascular dysfunction. At physiological levels, NO is essential for neuronal function, differentiation and survival through activation of signalling pathways that include the cyclic guanosine monophosphate (cGMP)/soluble guanylyl cyclase pathway [4] and S-nitrosylation, in which NO reversibly binds to thiol groups of proteins [5]. The vast network of NO-associated signalling paradigms further includes acetylation/deacetylation and methylation/demethylation modifications and peroxynitrite formation (leading to nitrotyrosination of protein residues), as well as modulation of gene expression via epigenetic changes [5–8]. How controlled S-nitrosylation/nitrotyrosination of proteins and activation of the NO/cGMP signalling pathway promote cellular survival and induce epigenetic changes while uncontrolled signalling promotes cell death and dysfunction remains to be elucidated. The aim of this Special Issue is to gather information encapsulating the above signalling pathways.

The articles published in this Special Issue largely cover (1) NO signalling in neuronal function and disease as well as (2) vascular targets in endothelial function and dysfunction, both of which involve the broad range of actions of this signalling molecule. In order to understand the contribution of NO to neuronal dysfunction, one has to consider that NO is a crucial molecule in cellular physiology. Numerous studies show the involvement of NO in neuronal development, plasticity, excitability, and transmission [8–12]. However, the common mechanisms across several neurodegenerative disorders relate to the neurotoxicity of NO and its downstream reactive nitrogen species (RNS). Enhanced nitrotyrosine immunoreactivity is evident in brains from patients with Alzheimer's and Parkinson's disease. Nitrated proteins are associated with *β*-amyloid deposition and nitrotyrosination of Tau protein and synaptophysin has been reported in brain samples from patients with Alzheimer's disease. The potential downstream signalling pathways of these posttranslational modifications are discussed in the review by S. A. Bradley and J. R. Steinert in this Issue. The cellular roles of NO in neurodegenerative disease, such as Alzheimer's, are reviewed by R. Balez and L. Ooi with a focus on neurotoxicity versus neuroprotection while the role of aberrant NO signalling in neurodevelopmental disorders such as Fragile X syndrome is investigated in the study by E. Lima-Cabello et al.

Other topics of this issue focus on aspects of vascular NO signalling with particular interest in fetoplacental dysfunction caused by abnormal eNOS regulation (as discussed by A. Leiva et al.). Endothelial function and eNOS regulation are essential for healthy cardiovascular responses but are also critical for adaptations during pregnancy. Several diseases associated with vascular dysfunction such as atherosclerosis, diabetes mellitus, hypertension, or preeclampsia involve altered NO signalling [13–15].

The paper by A. Leiva et al. reviews the mechanisms leading to abnormal NO signalling, which include reduced bioavailability of L-arginine (NOS substrate) and tetrahydrobiopterin (BH_4_), abnormal calcium-calmodulin signalling, and activation and inhibition of eNOS activity through phosphorylation of Ser^1177^ or Thr^495^, respectively [16]. NO has also been reported to play a crucial role in the transition of fetoplacental endothelial cells from a mitogenic to a metabolic phenotype in the macrocirculation, compared with a change from a metabolic to a mitogenic phenotype in the microcirculation in response to insulin in gestational diabetes mellitus [17]. Thus, the actions of NO are not only important during vascular adaptations to pregnancy and related dysfunctions but also essential during vascular signalling induced by physical training resulting in elevated nitrite and nitrate levels as reported in the study by A. M. Jacomini et al.

Together, this Special Issue highlights the tremendous diversity of NO signalling pathways with regard to its function in health and disease. All contributing publications have emphasised that NO represents an important signalling molecule and future research required in this field will extend the understanding of the broad actions of NO.

*Luis Sobrevia* *Luis Sobrevia* *Lezanne Ooi* *Lezanne Ooi* *Scott Ryan* *Scott Ryan* *Joern R. Steinert* *Joern R. Steinert*

## References

[1] Alderton W. K., Cooper C. E., Knowles R. G. (2001). Nitric oxide synthases: structure, function and inhibition. *Biochemical Journal*.

[2] Moncada S., Higgs E. A. (2006). The discovery of nitric oxide and its role in vascular biology. *British Journal of Pharmacology*.

[3] Palmer R. M. J., Ferrige A. G., Moncada S. (1987). Nitric oxide release accounts for the biological activity of endothelium-derived relaxing factor. *Nature*.

[4] Garthwaite J., Boulton C. L. (1995). Nitric oxide signaling in the central nervous system. *Annual Review of Physiology*.

[5] Nakamura T., Tu S., Akhtar M. W., Sunico C. R., Okamoto S.-I., Lipton S. A. (2013). Aberrant Protein S-nitrosylation in neurodegenerative diseases. *Neuron*.

[6] Hess D. T., Matsumoto A., Kim S.-O., Marshall H. E., Stamler J. S. (2005). Protein *S*-nitrosylation: purview and parameters. *Nature Reviews Molecular Cell Biology*.

[7] Steinert J. R., Chernova T., Forsythe I. D. (2010). Nitric oxide signaling in brain function, dysfunction, and dementia. *Neuroscientist*.

[8] Hardingham N., Dachtler J., Fox K. (2013). The role of nitric oxide in pre-synaptic plasticity and homeostasis. *Frontiers in Cellular Neuroscience*.

[9] Contestabile A. (2012). Role of nitric oxide in cerebellar development and function: focus on granule neurons. *Cerebellum*.

[10] Gamper N., Ooi L. (2015). Redox and nitric oxide-mediated regulation of sensory neuron ion channel function. *Antioxidants & Redox Signaling*.

[11] Steinert J. R., Robinson S. W., Tong H., Haustein M. D., Kopp-Scheinpflug C., Forsythe I. D. (2011). Nitric oxide is an activity-dependent regulator of target neuron intrinsic excitability. *Neuron*.

[12] Steinert J. R., Kopp-Scheinpflug C., Baker C. (2008). Nitric oxide is a volume transmitter regulating postsynaptic excitability at a glutamatergic synapse. *Neuron*.

[13] Guzmán-Gutiérrez E., Arroyo P., Salsoso R. (2014). Role of insulin and adenosine in the human placenta microvascular and macrovascular endothelial cell dysfunction in gestational diabetes mellitus. *Microcirculation*.

[14] Tousoulis D., Kampoli A.-M., Tentolouris C., Papageorgiou N., Stefanadis C. (2012). The role of nitric oxide on endothelial function. *Current Vascular Pharmacology*.

[15] Suslova T. E., Sitozhevskii A. V., Ogurkova O. N. (2015). Platelet hemostasis in patients with metabolic syndrome and type 2 diabetes mellitus: cGMP- and NO-dependent mechanisms in the insulin-mediated platelet aggregation. *Frontiers in Physiology*.

[16] Salsoso R., Guzmán-Gutiérrez E., Sáez T. (2015). Insulin restores l-arginine transport requiring adenosine receptors activation in umbilical vein endothelium from late-onset preeclampsia. *Placenta*.

[17] Westermeier F., Salomon C., Farias M. (2015). Insulin requires normal expression and signaling of insulin receptor A to reverse gestational diabetes-reduced adenosine transport in human umbilical vein endothelium. *The FASEB Journal*.

